# Beyond hemodynamics: environmental and psychosocial predictors of anxiety in emergency patients with gastrointestinal bleeding

**DOI:** 10.3389/fpsyg.2025.1668976

**Published:** 2025-10-22

**Authors:** Ramazan Kiyak, Gokhan Taskin

**Affiliations:** Department of Emergency Medicine, Faculty of Medicine, Balıkesir University, Balıkesir, Türkiye

**Keywords:** abdominal disease, anxiety, emergency treatment, gastrointestinal bleeding, public health

## Abstract

**Purpose:**

We investigated the state anxiety level and the factors affecting it in patients with Gastrointestinal Bleeding (GIB) who were followed up in the Emergency Department (ED).

**Material-methods:**

The cross-sectional study was conducted with 107 patients (62 females, 45 males) who were admitted to the ED of Balikesir University Faculty of Medicine Hospital between 01.02.2025–10.05.2025 and diagnosed with GIB using the complete census method. In the study in which a correlational screening model was used, data were collected with the Demographic Information Form and the State Anxiety Inventory. Descriptive statistics, independent samples *t*-test, and One-way ANOVA tests were applied in the analysis.

**Results:**

When the results of the study are examined, it is seen that the state anxiety levels of the patients followed up in the ED with the diagnosis of GIB are at a moderate level; there is no significant difference between state anxiety and gender, occupation, marital status and previous bleeding; and there are significant differences between state anxiety and being disturbed by the monitor machine sound in the environment of ED, being disturbed by the environment, being disturbed by the stretcher, being disturbed by the crowd, being disturbed by seeing other patients and being disturbed by not having physical communication with the outside. No statistically significant difference was found according to educational status.

**Conclusions:**

It can be said that reducing the noise and chaos in the ED and providing a calmer and more supportive environment for the patient can reduce the state anxiety levels of patients with GIB and similar acute conditions and thus positively affect both their psychological wellbeing and medical outcomes. These findings may inform the development of targeted interventions—such as environmental modifications, structured patient education, or supportive practices—that can be implemented in the ED to enhance patient care.

## 1 Introduction

Gastrointestinal Bleeding (GIB) is a life-threatening clinical condition with high morbidity and mortality and is frequently encountered in the Emergency Department (ED). The clinical presentation may also influence patients' anxiety; for example, visible hematemesis in upper GIB often provokes more immediate fear and discomfort compared to melena in lower GIB. In the US, approximately 300,000 patients are hospitalized, and up to 30,000 patients die each year due to upper GI bleeding. Massive upper GIB can develop hemorrhagic shock in a short time, and the patient's life is at serious risk (Hu et al., 2024). The clinical severity of GIB, which requires urgent intervention, causes not only physical but also psychological problems for patients.

It is known that GIB experienced in emergency conditions causes significant anxiety in patients (Adarsh and Kiran, 2014). In addition, various studies have shown that GIB patients requiring invasive diagnosis and treatment have increased anxiety levels (Ghonaem and Ibrahim, 2019; Uçaner et al., 2024). Felemban et al. (2024) reported that even before elective upper gastrointestinal endoscopy, the state anxiety level increased significantly in approximately half of the patients. In patients undergoing emergency GIB, it is thought that anxiety levels will increase even more due to the fear of encountering a life-threatening situation, the uncertainty of the emergency room environment, and fears about the interventions to be performed (emergency endoscopy, blood transfusion, etc.). Although endoscopy is usually performed under sedation and, therefore, less likely to cause discomfort during the procedure itself, the anticipation of such an intervention may still contribute to patient anxiety. As a matter of fact, it has been shown that patients who do not know enough about the disease and the procedures to be performed have higher anxiety (Felemban et al., 2024). Patients' generally low level of knowledge about GIB also reinforces this concern (Hu et al., 2024). In the literature, it has been reported that young patients and women report higher anxiety in such acute situations, and factors such as lack of information may increase anxiety (Felemban et al., 2024; Karpuzcu et al., 2025). Therefore, it is important to examine the demographic and clinical factors determining the level of anxiety in patients presenting to the ED with GIB.

High levels of anxiety are not only limited to the psychological state of the patient but can also negatively affect the physiologic course and treatment. Increased sympathetic nervous system activity and catecholamine release in acute stress may lead to tachycardia and hypertension and accelerate ongoing bleeding (Hu et al., 2024; Kaye et al., 2022). However, it should be noted that tachycardia in patients with gastrointestinal bleeding can also result from intravascular volume changes, and in patients receiving beta-blockers, the typical tachycardic response may be blunted. In addition, this hyperadrenergic state triggered by anxiety may cause changes such as increased respiratory rate, increased pain perception, and muscle tension, making it difficult to perform medical interventions. Studies show that problems such as difficulty in cooperation, increased discomfort, and inability to tolerate treatment are more common in patients with high anxiety (Karpuzcu et al., 2025). For these reasons, not only hemodynamic stabilization but also evaluation of the psychological status of patients with GIB in the ED and, if possible, improvement of their psychological status is an integral part of the holistic approach.

In this study, it was aimed to measure the state anxiety levels of adult patients followed up in the ED due to GIB using the State Anxiety Inventory (STAI) and to analyze the factors that may affect these anxiety levels. Thus, the effects of an acute life-threatening clinical condition on patient psychology and the factors determining these effects will be better understood and will contribute to the literature on the role of psychological support in the management of GIB in the ED.

## 2 Method

### 2.1 Research model

This study was designed based on the relational screening model, which is one of the quantitative research approaches, in order to examine the state anxiety level of patients followed up in the ED with the diagnosis of GIB and the factors affecting it. The relational screening model is a research method to determine the relationship between more than one variable (Büyüköztürk et al., 2008).

### 2.2 Research group

This research is a cross-sectional study and the complete census method was used as the sampling method. A complete census is an effective data collection method that eliminates sampling error since the entire population is reached (Arikan, 2007). A total of 107 individuals who were admitted to the ED of Balikesir University Faculty of Medicine Hospital during the 3 months between 01.02.2025 and 10.05.2025 and diagnosed with GIB as a result of the tests performed were included in the study. Although the complete census method was applied to avoid sampling bias, the short study period may limit the representativeness of the sample compared to other hospitals or national populations. Therefore, the findings should be interpreted as reflecting the experience of a single tertiary care center within a limited timeframe. Of the participants, 62 (57.9%) were female and 45 (42.1%) were male. Findings regarding the demographic characteristics of the participants are presented in Table 1.

**Table 1:** Demographic information of the participants.

**Variables**		**X̄**	**S.D**.
Age		57.68	18.07
		**n**	**%**
Gender	Woman	62	57.9
	Male	45	42.1
Education status	Primary education	42	39.3
	High school	25	23.4
	University	34	31.8
	Postgraduate	6	5.6
Profession	Working	38	35.5
	Not working	69	64.5
Marital status	Single	29	27.1
	Married	78	72.9
Previous bleeding status	Yes	38	35.5
	No.	69	64.5
Disturbance from monitor and medical devices noises	Yes	39	36.4
	No.	68	63.6
Disturbance from the environment	Yes	35	32.7
	No.	72	67.3
Being uncomfortable in a stretcher	Yes	28	26.2
	No.	79	73.8
Discomfort in crowds	Yes	42	39.3
	No.	65	60.7
Discomfort of seeing other patients	Yes	37	34.6
	No.	70	65.4
Feeling uncomfortable not having physical contact with the outside	Yes	34	31.8
	No.	73	68.2
	**Total**	107	100

### 2.3 Data collection tools

This study was ethically approved by the Balikesir University Health Sciences Non-invasive Research Ethics Committee with the decision numbered 2025/134. The research was conducted following the guidelines of the revised Declaration of Helsinki.

#### 2.3.1 Personal information form

The personal information form prepared by the researchers to determine the personal information of the participants consisted of two parts. The first part included 6 personal questions about the participants' “age, gender, educational status, professional status, marital status and whether they have had bleeding before”. The second part consisted of six questions about the environmental factors of the ED environment, including “being disturbed by the sound of the monitors and medical devices, being disturbed by the environment, being disturbed by the stretcher, being disturbed by the crowd (referring to the overall patient density in the ED, not relatives or outsiders), being disturbed by seeing other patients, and being disturbed by not having physical communication with the outside”. It should be noted that the actual sound level (decibel) was not objectively measured; disturbance was recorded based on patients' subjective perception. Although alarm volumes can technically be adjusted, the cumulative effect of multiple devices and the overall ED noise environment may still create significant distress. Similarly, patient discomfort regarding long waiting times was assessed subjectively, and the exact duration in minutes or hours was not recorded.

#### 2.3.2 State anxiety inventory

The State Anxiety Inventory was developed by Spielberger and Gorsuch in 1964 to measure the level of state anxiety in normal and abnormal individuals. Turkish adaptation, validity, and reliability studies were conducted by Öner and Le Compte (1983). The scale consists of 20 items. The items in the scale are in a 4-point Likert-type. 1 means “not at all” and 4 means “completely”. Scores obtained from the scale theoretically range between 20 and 80. Scale items consist of direct (straight) and inverted statements. When reverse statements expressing positive emotions are scored, those with a weight of 1 are converted to 4, and those with a weight of 4 are converted to 1. In direct statements expressing negative emotions, responses with a value of 4 indicate high anxiety, while responses with a value of 1 in reverse statements indicate low anxiety. In inverse statements, responses with a value of 4 indicate low anxiety, while responses with a value of 1 indicate high anxiety. In the State Anxiety Inventory, 10 items (1, 2, 5, 8, 10, 11, 15, 16, 19, and 20) are inverse statements (Spielberger et al., 1971). The Cronbach alpha value of the scale adapted into Turkish by Öner and Le Compte (1983) was determined as 0.94. In this study, Cronbach's alpha value was found to be 0.91.

The following formula was used to characterize the averages obtained from the state anxiety inventory.


$$\begin{array}{l} \text{State Anxiety Level Score Range}=\frac{Max.\;Score-Min.\;Score}{Likert\;Type}\\ \;=\frac{80-20}{4}=15\;Score\; \end{array}$$

20–35 score = Low Level Anxiety

36–50 score = Moderate Anxiety

51–65 score = High Level Anxiety

66–80 score = Very high level of anxiety.

### 2.4 Data analysis

The IBM SPSS 27 package program was used to analyze the data obtained. Skewness and Kurtosis values were taken into consideration for the normality test of the data and it was accepted that they showed normal distribution since these values were in the range of −1 to +1 (Alpar, 2016; Başol et al., 2019). In the analysis of the data obtained, descriptive statistics (percentage, frequency, minimum, maximum, mean and standard deviation) as well as independent samples *t*-test for pairwise comparisons and One-way ANOVA for comparisons of more than two groups were used. Significance was accepted as *p* < 0.05.

### 2.5 Ethical approval

Before starting the research process, ethics committee approval was obtained from Balikesir University Health Sciences Non-Interventional Research Ethics Committee with the number 2024/210 and date 03/12/2024. Before the research, the importance of the research was explained to the patients with GIB, and informed consent form was obtained.

## 3 Results

The mean score of the participants from the state anxiety inventory was found to be (X̄ = 47.34) (Table 2). This average shows that the anxiety levels of the participants during GIB were at a moderate level.

**Table 2:** Mean scores of the participants from the state anxiety inventory.

**Scales**	**N**	**Min**.	**Max**.	**X̄**	**S.D**.
State anxiety inventory	107	32	69	47.34	9.71

When Table 3 was examined, it was seen that the state anxiety levels of the participants do not show a significant difference according to gender (*p* = 0.566). According to occupational status, although it was observed that the anxiety scores of employed individuals were higher, this difference was not at a statistically significant level (*p* = 0.071). Similarly, there was no significant difference in anxiety levels according to marital status (*p* = 0.470) and previous bleeding (*p* = 0.925). When evaluated in terms of environmental factors, statistically significant differences were found in terms of being disturbed by the sound of monitors and medical devices (*p* < 0.001), being disturbed by the environment (*p* < 0.001), being disturbed by the stretcher (*p* < 0.001), being disturbed by the crowd (*p* < 0.001), being disturbed by seeing other patients (*p* < 0.001) and being disturbed by not being able to physically communicate with the outside (*p* < 0.001). According to these results, it was determined that the state anxiety levels of individuals who were disturbed by the environmental stress sources were significantly higher. Although no significant difference was observed in the analysis according to educational level (*p* = 0.060), it is noteworthy that the anxiety scores of individuals with university and graduate education are higher than the other groups. These findings reveal that demographic characteristics have a limited effect on state anxiety, whereas environmental stress factors significantly affect the level of anxiety.

**Table 3:** Comparison of participants' state anxiety levels according to demographic characteristics.

**Scale**	**Variables**	**N**	**X̄**	**S.D**.	**t**	**p**
State anxiety inventory	**Gender**					
		Woman	62	46.87	9.53	0.576	0.566
		Male	45	47.98	10.02		
		**Profession**					
		Working	38	49.79	10.98	1.840	0.071
		Not working	69	45.99	8.73		
		**Marital status**					
		Single	29	46.41	6.81	−0.726	0.470
		Married	78	47.68	10.61		
		**Previous bleeding status**					
		Yes	38	47.45	8.03	0.094	0.925
		No.	69	47.28	10.57		
		**Disturbance from monitor and medical devices noises**					
		Yes	39	53.31	10.62	5.425	< 0.001^*^
		No.	68	43.91	7.25		
		**Discomfort from the environment**					
		Yes	35	54.80	10.59	6.55	< 0.001^*^
		No.	72	43.71	6.80		
		**Uncomfortable with the stretcher**					
		Yes	28	52.61	9.47	3.518	< 0.001^*^
		No.	79	45.47	9.14		
		**Discomfort in crowds**					
		Yes	42	53.26	10.45	5.804	< 0.001^*^
		No.	65	43.51	6.94		
		**Discomfort of seeing other patients**					
		Yes	37	53.65	11.23	5.529	< 0.001^*^
		No.	70	44.00	6.81		
		**Feeling uncomfortable not having physical contact**					
		**with the outside**					
		Yes	34	52.06	10.71	3.625	< 0.001^*^
		No.	73	45.14	8.41		
		**Education status**	**N**	**X̄**	**S.D**.	**F**	**p**
		Primary education	42	44.45	7.73	2.547	0.060
		High school	25	47.52	9.55		
		University	34	50.21	11.21		
	Postgraduate	6	50.50	9.71		

^*^p < 0.05.

## 4 Discussion

The findings obtained in this study, which examined the state anxiety level and the factors affecting it of patients followed up in the ED with the diagnosis of GIB, were compared and interpreted with similar studies in the literature.

State anxiety levels of patients followed up in the ED with a diagnosis of GIB were found to be moderate. However, while high anxiety levels are expected in individuals who face an acute and life-threatening clinical picture, the results obtained were below this expectation. This may be explained by various factors such as the level of acceptance of the current clinical situation, previous similar experiences or the approach of the healthcare personnel. A review of the literature revealed that patients hospitalized in the ED with a diagnosis of myocardial infarction or COVID-19 pneumonia had significantly higher state and trait anxiety scores compared to healthy controls. Moreover, anxiety levels were found to be similar in patients with COVID-19 and myocardial infarction (Çaglar and Kaçer, 2022). This result may be due to the fact that, contrary to expectations, anxiety levels in patients followed up in the ED with a diagnosis of GIB remained at a moderate level, and the perceived threat level in GIB patients was lower or reduced by factors such as patients' acceptance of the situation, previous experiences and effective approach of healthcare personnel, compared to diseases such as myocardial infarction and COVID-19 pneumonia, where the perception of direct risk of death is more prominent.

According to the findings of the study, no significant difference was found between the state anxiety levels of patients with GIB according to gender, occupation, marital status, previous bleeding, and educational status. This may be due to the individual coping mechanisms of anxiety levels, the severity of the current clinical condition, and the patients' prior experiences in accessing health services. When the literature is examined, there is no consensus on the effect of sociodemographic factors on anxiety. For instance, some studies have reported that women and younger patients tend to experience higher levels of anxiety in acute medical conditions (Felemban et al., 2024; Lauriola et al., 2019), whereas other studies found no such associations (Paniyadi et al., 2019). Cultural differences, variability in clinical settings, and differences in measurement tools may explain these inconsistencies. In our study, the acute and potentially life-threatening nature of GIB might have overridden the influence of sociodemographic variables, leading to relatively uniform anxiety levels across subgroups. This suggests that situational and environmental stressors in the ED environment may play a more dominant role than demographic characteristics in shaping patients' anxiety responses. However, different patient populations and clinical conditions may change the results. In a study conducted in India, although all patients experienced mild to moderate anxiety, no statistical difference was found between age, gender or education level and anxiety severity (Paniyadi et al., 2019). On the other hand, in a study conducted in patients undergoing endoscopy, it was found that female patients undergoing endoscopy for the first time experienced significantly higher anxiety and distress than male patients with previous endoscopy experience (Lauriola et al., 2019). Thus, it can be said that the effect of sociodemographic variables on anxiety levels may vary according to contextual factors, clinical experiences, and individual differences. This situation reveals that each patient group should be evaluated individually, and the factors affecting anxiety levels should be addressed with a multidimensional approach rather than a singular approach.

Significant differences were found between environmental stressors in the ED environment and state anxiety. In particular, it was observed that the anxiety scores of individuals who were disturbed by environmental factors such as noise and crowding were significantly higher. The mean difference between groups often reached 7–10 points on the STAI scale, which represents a shift from moderate anxiety toward the high anxiety range. From a clinical perspective, such an increase is not trivial: higher anxiety levels may trigger sympathetic activation, worsen hemodynamic parameters such as blood pressure and heart rate, and reduce patient cooperation during urgent interventions. Moreover, patients experiencing discomfort due to stretcher use or inability to communicate with the outside world may feel a greater sense of vulnerability and isolation, which can reduce tolerance of prolonged observation and negatively affect satisfaction with care. Therefore, even modest improvements in the ED environment—such as reducing monitor alarm volume, optimizing patient flow to prevent crowding, or providing more comfortable stretchers—could translate into meaningful reductions in patient anxiety and potentially better clinical outcomes. In ED, monitor alarms, medical device sounds and background noise from staff and other patients are continuous. In the literature, it is stated that noise in the hospital environment increases anxiety and sleep disorders, especially in units such as intensive care and operating rooms (Delaney et al., 2019). The findings of this study also revealed that the anxiety levels of patients who reported discomfort from monitor and device sounds were higher than those who stated that they were not affected by these sounds. In addition, the crowdedness of the ED, increased noise and prolonged waiting times are other important factors that increase anxiety. Although our study specifically examined patients with GIB, it should be emphasized that such environmental stressors are likely to provoke anxiety across a wide range of ED populations, underscoring the need for general environmental modifications aimed at improving patient wellbeing. Within the scope of the study, it was found that the anxiety scores of individuals who were “disturbed by the crowdedness of the environment” were statistically significantly higher than those who were not disturbed. Similarly, Wang et al. (2020) reported that crowd-related variables such as patient density and waiting time in the ED were positively correlated with anxiety levels. Accordingly, it is understood that anxiety levels increase significantly in individuals who have to wait for a long time in the ED and who are surrounded by a large number of patients. From a translational perspective, interventions such as establishing designated quiet bays for vulnerable patients, relocating high-noise equipment, or implementing patient flow strategies to reduce visible crowding may represent practical approaches to mitigate these stressors in daily ED practice. Another source of environmental stress faced by GIB patients is the discomfort caused by the physical environment. It was determined that the anxiety levels of individuals who were uncomfortable lying on a stretcher, who felt uneasy seeing other patients around them, or who complained about not being able to communicate with the external environment were significantly higher than those who did not define these situations as a problem. Lying on a stretcher may create a feeling of vulnerability and loss of control. Waiting on a stretcher, especially in the corridor of the ED, may cause both physical discomfort and a sense of isolation from the environment. The literature shows that patients who wait on a stretcher in emergency corridors feel worse and their satisfaction with healthcare services decreases (Chang et al., 2016). Patients feeling disconnected from the outside world is another common anxiety factor in the environment of ED. These units are mostly closed, deprived of natural light and where telephone communication may be limited. Lack of social support and disconnection from the outside world increase anxiety by reinforcing feelings of loneliness and helplessness. Similar to the findings of the study, a study conducted by Gheshlaghi et al. (2021) showed that the presence of a family member with the patient during the invasive procedure significantly reduced anxiety levels. In addition, seeing other patients is also an important source of stress for GIB patients. In particular, witnessing patients in serious condition or bleeding can lead to more intense anxiety about one's own health status. In qualitative studies, it is reported that the theme of “fear and anxiety against uncertainty and unknownness” is frequently expressed in relation to the environment of ED (Mutlu et al., 2021). In this context, it is understandable that individuals who see patients with similar or more severe conditions around them experience anxiety, especially in the patient group in the study.

According to the results of the study, it was observed that the state anxiety levels of the patients who were followed up in the ED with the diagnosis of GIB were at a moderate level. Moreover, while there was no significant difference between the state anxiety levels of patients with GIB according to gender, educational status, occupation, marital status and previous bleeding, it was found that there were significant differences between the state anxiety levels of patients with GIB according to being disturbed by the monitor and medical devices sound in the emergency room environment, being disturbed by the environment, being disturbed by the stretcher, being disturbed by the crowd, being disturbed by seeing other patients and being disturbed by not having physical communication with the outside. It can be said that reducing the noise and chaos in the ED and providing a calmer and more supportive environment for the patient may reduce the anxiety levels of patients with GIB and similar acute conditions and thus positively affect both their psychological wellbeing and medical outcomes. Although this study focused on patients with gastrointestinal bleeding to ensure clinical homogeneity, the environmental stressors identified are not unique to this group. Replication in other ED populations undergoing urgent interventional procedures (e.g., chest tube insertion, cardioversion, central venous catheterization) would be valuable to test generalizability and explore procedure-specific influences on anxiety.

Incorporating anxiety assessment into routine ED care for GIB patients appears feasible using an ultra-brief, staged approach: a rapid triage screen (e.g., STAI-6 or a 0–10 visual analog anxiety scale), a repeat check after initial stabilization, and EHR-embedded thresholds (e.g., VAS-A ≥7) that trigger simple, low-cost interventions (quiet areas, safe alarm reduction, brief guided breathing, optional family presence, and structured information). Documenting screens and communicating high-anxiety flags during handoff may improve cooperation with procedures and patient experience without adding substantial workload.

Future research can prospectively evaluate the effect of environmental improvements and anxiety-relieving interventions on patient outcomes. Evidence from other acute care settings suggests that creating designated quiet zones, reducing unnecessary monitor alarms, and optimizing patient flow can significantly reduce stress. Furthermore, interventions such as allowing family presence during certain procedures, structured patient information and counseling, and non-pharmacological methods including music therapy, guided breathing, or relaxation techniques have all been associated with reductions in anxiety levels. Although implementing music therapy in a busy ED can be challenging, practical adaptations such as allowing patients to use personal headphones or providing short relaxation audio during waiting times may offer feasible, low-cost options without disrupting clinical care Incorporating such approaches into ED practice may not only improve patient wellbeing but also enhance cooperation with medical procedures and overall satisfaction with care. Thus, it is thought that by developing holistic emergency care approaches, both the management of life-threatening situations and the experiences of patients can be improved.

## 5 Conclusion

Our findings provide valuable insight into an under-addressed aspect of emergency care. By identifying the presence of significant anxiety and its correlates in patients with GIB, the study contributes to a more holistic understanding of patient care in the ED. These insights can inform future investigations and encourage emergency physicians to consider the psychological wellbeing of patients alongside their immediate medical needs, ultimately helping to improve patient experience and care outcomes. In practical terms, implementing strategies such as reducing environmental noise, establishing quiet zones, facilitating family presence, and using simple relaxation interventions (e.g., music therapy, breathing exercises) could provide low-cost, evidence-based ways to alleviate anxiety in patients with GIB and similar acute conditions.

## 6 Limitations

This study has several limitations that warrant consideration. First, it was conducted at a single center with a relatively short study period, which may limit the generalizability of the findings to other hospital settings, different regions, or national populations. Although we applied the complete census method to minimize sampling bias, the restricted timeframe may not fully capture seasonal variations or institutional differences in patient characteristics. Second, the cross-sectional design captures patient anxiety at one point in time and thus cannot establish causality or determine how anxiety levels might change over the course of evaluation and treatment. Third, the sample size was relatively modest, which may reduce the statistical power to detect smaller differences and limit the robustness of subgroup analyses. Finally, no a priori power analysis was performed, which restricts our ability to determine whether the study was adequately powered to detect all clinically relevant associations. Future multi-center studies with larger sample sizes and formal power calculations are recommended to validate and extend these findings.

## Data Availability

The raw data supporting the conclusions of this article will be made available by the authors, without undue reservation.
